# Microscopic Positive Tumor Margin Increases Risk for Disease Persistence but Not Recurrence in Patients with Stage T1-T2 Differentiated Thyroid Cancer

**DOI:** 10.1155/2020/5287607

**Published:** 2020-01-10

**Authors:** Olfat Kamel Hasan, Sarah De Brabandere, Irina Rachinsky, David Laidley, Danielle MacNeil, Stan Van Uum

**Affiliations:** ^1^Department of Medicine/Diagnostic Imaging, Hamilton Health Sciences, McMaster University, Hamilton, Canada; ^2^Departments of Diagnostic Imaging, Division of Nuclear Medicine, Western University, London, Ontario, Canada; ^3^Departments of Otolaryngology-Head and Neck Surgery, Western University, London, Ontario, Canada; ^4^Departments of Medicine, Schulich School of Medicine and Dentistry, Western University, London, Ontario, Canada

## Abstract

**Introduction:**

Differentiated thyroid cancer (DTC) has an overall excellent prognosis. Patients who develop recurrent disease have a more unfavorable disease course than those with no recurrence. Higher recurrence rates are seen with incomplete surgical resection and gross positive margins. It is unclear whether microscopic positive margin affects disease recurrence rates as much as grossly positive margin. *Aim of the Study*. To assess whether microscopic positive margin is an independent predictor of disease recurrence in patients with overall low-risk DTC. *Patients and Methods*. We conducted a retrospective single-center institutional review of 1,583 consecutive patients' charts from 1995–2013 using the Canadian Thyroid Cancer Consortium Registry. We included adult patients with nonmetastasizing T1 and T2 DTC with a minimum of three years follow-up. Univariate and multivariate analyses were used to study factors that may influence the risk of persistent/recurrent disease. Strict definitions of persistent versus recurrent disease were applied.

**Results:**

963 patients (152 men and 811 women) were included in the study with a mean age of 46 years. Microscopic positive margins were present in 12% of the specimens and were associated with an increased rate of persistent disease (8% versus 2% in the controls) but not with an increased risk of recurrent disease (1% in both groups). T2 tumors had a significantly higher incidence of positive margins than T1 tumors (48% versus 36%) and significantly higher nodal staging.

**Conclusions:**

Microscopic positive margin in the histopathology report in patients with low-risk DTC was associated with a higher rate of persistent disease but did not increase the risk of disease recurrence. A close follow-up of biomarkers and occult residual cancerous lesions is needed, especially in the first year. Further studies are needed to determine whether additional therapeutic measures to prevent recurrence are indicated in T1 and T2 DTC with positive microscopic surgical margins.

## 1. Introduction

Differentiated thyroid cancer (DTC) is the most common endocrine malignancy with an increasing incidence in the last several decades [1, 2]. Most patients with DTC have an excellent overall prognosis with a 10-year disease-free survival rate of 90–97% [3, 4]. However, recurrence of thyroid cancer is not unusual and occurs in 2 to 20% of patients, depending on the study cohort, disease staging, and variations in definitions of recurrence versus persistent disease [5–7]. Recurrence risk is low occurring in 1.4–3% and has a significant impact on mortality [6, 8]. It is well known that patients who develop recurrent disease after curative treatment have a higher probability of death resulting from thyroid cancer [3]. Therefore, it is imperative to stratify risk and to identify patients who are more prone to recurrence as this could potentially affect clinical management.

Several risk factors are associated with a higher recurrence rate, including increased age at diagnosis, larger tumor size, aggressive tumor subtypes, multifocal disease, extrathyroidal extension, and positive surgical margins. The modified 2015 American Thyroid Association (ATA) risk stratification system identifies the completeness of surgical resection with negative surgical margins as an important determinant of the outcome since positive margins are generally associated with an intermediate or high risk of recurrence [9]. However, most patients with positive margins do not have gross residual disease but can rather have microscopic positive margins. There has been conflicting data in the literature on the clinical relevance of microscopic positive margins on final pathological analysis in relation to the risk of recurrence in DTC. Hong et al. [10] found that microscopic positive surgical margins have a significantly higher incidence of early recurrence, but other studies did not find such a relationship [7, 11, 12]. It is unclear whether microscopic positive margins affect disease persistence or recurrence rates to the same extent as grossly positive margins.

Importantly, prior studies did not provide clear definitions for disease persistence or recurrence, so outcomes cannot be separated between these two entities. Sapuppo et al. [13] recently analyzed a large consecutive series of patients with DTC to evaluate disease prevalence and outcomes. This group used clear definitions for “recurrent” disease (relapse after being 12 months disease free) and “persistent” disease (present ab initio since diagnosis). They reviewed a total of 639 of 4292 (14.9%) patients who had disease events after initial treatment, most (78%) with persistent disease and 22% with recurrent disease. It was found that male sex, age, follicular subtype, T status (T3; OR = 3), and N status (N1b; OR = 7.7) were independently associated with persistent disease status; only N status (N1b; OR 7.7) was associated with recurrent disease. They concluded that in patients with DTC after initial treatment, persistent disease is more common and has worse prognosis than recurrent disease. Therefore, in the current study, we established rigorous definitions for persistent and recurrent disease that were applied to extract separate outcomes.

Radioactive iodine (RAI) administration is an established method of remnant thyroid tissue ablation or therapy. According to the 2015 ATA guidelines, RAI is not routinely recommended for low-risk patients but may be considered for intermediate risk patients and is indicated for high-risk DTC patients [9]. Since microscopic positive margins have unclear implications on recurrence, patients may be overtreated with adjuvant therapies such as RAI. On the other hand, if the recurrence risk is not increased in patients with microscopic positive margins and there are no other aggressive features, then this group does not require RAI treatment. Therefore, the main objective of our study was to determine whether the presence of microscopic positive margin is an independent predictor of disease recurrence in patients with low-risk DTC and if this could inform the decision whether or not to use RAI.

## 2. Patients and Methods

### 2.1. Patients

We performed a retrospective single-center review of patients included in London, Ontario, which is a site of the Canadian Thyroid Cancer Consortium Registry (CTCCR), from 1995 to 2013. The CTCCR is a large thyroid cancer registry that collects data on patients with DTC. For this study, we evaluated patients who were referred to the London, Ontario, thyroid cancer clinic from the Southwestern Ontario region. The database was established in 2005 and was used to collect data related to the participants' thyroid cancer presentation, management, and outcomes (retrospectively for patients diagnosed before 2004 and prospectively thereafter). Ethics approval for this study was granted by the Western University Research Ethics Board. Each patient in the registry consented to have their deidentified data collected and analyzed.

Patients were included if they were 18 years or older at the time of diagnosis, had differentiated (papillary and/or follicular) thyroid cancer, stage T1 or T2 (TNM staging, AJCC 7th edition) with or without nodal involvement, were diagnosed between 1995 and 2013, and had a minimum follow-up of 3 years. Patients were excluded if they had not undergone a total thyroidectomy (one or two stages within 6-month maximum interval), had gross residual disease, or had distant metastases at presentation.

We collected the following variables for each patient: age at diagnosis, gender, date of diagnosis, histology (papillary vs. follicular), primary tumor size, TNM stage, type of surgery (single total thyroidectomy vs. two-part thyroidectomy), lymph node dissection and extent (central, lateral, or mediastinal), administration of RAI and I-131 dose, disease outcomes, last follow-up date, status at last follow-up, and death, including the cause of death (related or unrelated to disease). Patients with recurrent or persistent disease were reviewed in detail, and additional data were collected on the location (local or distant) and the mode of diagnosis of recurrence and persistent disease (biochemical, imaging per modality, clinical, and/or biopsy).

We reviewed 1,583 consecutive T1/T2 patients who underwent total or complete thyroidectomy for DTC between 1995 and 2013. We excluded 620 patients: 415 patients had follow-up for less than 3 years, 117 patients had incomplete data, and 85 were excluded for one or more of the following reasons: T3 staging (after re-checking), distant metastases at diagnosis, age less than 18 years old at the time of diagnosis, and/or a pathology diagnosis other than papillary or follicular. Two patients actually had recurrent disease at the time of presentation at our center, and one patient was excluded because the completion thyroidectomy was done more than 6 months apart from the first hemithyroidectomy. The final number of patients included was 963.

### 2.2. Clinical Definitions

We used the following clinical and pathology definitions.

  Disease-free: the absence of structural disease on imaging and negative biomarkers (nonstimulated thyroglobulin (TG) <2 ng/ml and stimulated TG <10 with negative anti-TG antibodies), with a negative stimulated TG test one year after initial treatment (total thyroidectomy plus/minus radioactive iodine therapy), if available. Cutoff TG values were selected based on the available lab measurements during the study period over 2 decades.
  Persistent disease: the absence of normalization of biomarkers (TG remaining >2 ng/ml, and/or stimulated TG >10 ng/ml, and/or positive anti-TG antibodies) and/or the presence of structural disease on imaging studies (US or cross-sectional, or RAI scan) after at least 12 months after initial treatment.
  Recurrence: disease recurrence could be structural, biochemical, or both. Structural recurrence was defined as local thyroid bed recurrence, lymph node recurrence, or distant metastases and was diagnosed by either positive biopsy or findings highly suggestive on imaging. Biochemical recurrence was defined as a consecutive increase in TG and/or TG antibodies that was sufficient to convince the expert clinician to diagnose recurrence. Diagnosis of recurrence of DTC required a preceding disease-free period, as defined above.
  Disease progression: disease progression could be structural, biochemical, or both. Structural progression was based on the enlargement of previously known lesions and/or development of additional new lesions confirmed by imaging and/or positive biopsy. Biochemical progression was defined as an increase in biomarkers by more than 25% from the baseline over at least three consecutive readings. The baseline measurement was the TG value measured after surgery prior to administration of RAI and before the effect of RAI therapy.
  Persistent improving disease: this was defined as a reduction in biomarkers by more than 25% from the baseline measurement (over three consecutive readings) without normalization and stable or improving structural disease.
  Persistent stable disease: a fluctuation in biomarkers within 25% from the baseline measurement with no significant changes in structural dimensions.

Of note, for all definitions, in case of uncertainty, we relied on expert clinical judgment.

  Positive margins based on pathology: a margin is only called positive when the tumor extends to the peripheral inked specimen margin, i.e., the tumor must have ink on its surface. It is not called positive if it is 0.01 mm from the inked margin. If the margins are close but no tumor is seen at the inked margin, it is defined as a negative margin. A tumor can extend to the inked margin and is not extrathyroidal. Similarly, a tumor can extend beyond the thyroid tissue (extrathyroidal extension) but not to the inked specimen margin. This depends on how much tissue is removed surgically beyond the thyroid gland.

### 2.3. Pathological Analysis Protocol

The London Health Science Center follows a very strict protocol for the analysis of thyroidectomy specimens. When receiving a specimen in the pathology lab, it is inked and oriented (i.e., anterior surface black, posterior surface blue, and isthmus margin orange if a hemithyroidectomy). After inking, the specimen is serially sectioned in the transverse (axial) plane from superior to inferior at 3–4 mm interval thickness. Up to 20 cassettes will usually be submitted for a hemithyroidectomy, and in most cases, all of the tissue can be submitted in these 20 cassettes. The superior and inferior poles are cut in the sagittal plane. If it is an abnormally large hemithyroidectomy, it is usually submitted in 20 cassettes, thereby sampling all of the different identified nodules. If a well-encapsulated lesion is identified, the entirety of the capsule will be submitted as it is critical to assess the presence of transcapsular invasion or capsular lymphovascular invasion. For total thyroidectomies, all of the tissue is submitted into a minimum of 20 cassettes, thereby sampling all of the identified nodules. If more cassettes are required, then more are submitted. Well-encapsulated nodules in a total thyroidectomy are treated as they are in hemithyroidectomies. The status of the resection (inked) margins should be reported as “involved-positive” or “uninvolved-negative” with a tumor in the final report.

### 2.4. Statistical Analysis

Descriptive statistics were used to present the study variables. Mean values and standard deviations were calculated for normally distributed continuous variables; median and range values are presented for nonnormally distributed continuous variables. A chi-squared test was used to compare categorical variables. A one-way ANOVA test was conducted to compare continuous variables. Multivariate Cox regression analysis was performed using all variables that were significant in the univariate analysis using SPSS version 25 (IBM Corporation, Armonk, NY, USA). A *P* value of <0.05 was used to indicate statistical significance.

## 3. Results

Baseline characteristics of the participating patients are shown in Table 1. Sixteen percent (16%) of the patients were male and 84% were female, with a mean age of 46 ± 13 years. Sixty-five percent (65%) underwent a one-stage total thyroidectomy, and 35% underwent a two-stage thyroidectomy. Central neck dissection was performed in 9%, and lateral neck dissection was done in 4%. Most patients (96%) had papillary thyroid cancer and 4% had follicular thyroid cancer. Sixty-three percent (63%) of patients had a T1 tumor and 37% had a T2 tumor. Lymph node involvement was found in 15% of patients.

Microscopic positive margins were found in 12% of patients (112 patients), while 88% (851 patients) had clear margins. There was no statistically significant difference for sex, age at presentation, initial thyroid surgical management, initial neck dissection, or histological type of thyroid cancer between patients with positive versus clear margins. Positive margins were found in 54 out of 360 (15%) patients with T2 tumors which was significantly higher than the 58 out of 603 patients (9.6%) of all patients with T1 tumors (*P*=0.026) (Table 2).

Nodal involvement (N) categories were used for staging, as can be seen in Table 1. Nx/N0 denotes negative nodal staging, whereas N1a/N1b denotes positive nodal staging (16). N Positive nodal staging (N1a/N1b) was also found more frequently in patients with positive margins (26/112 patients, 23%) than in patients with clear margins (116/851 patients 14%, *P*=0.024).

### 3.1. Initial RAI Ablation/Therapy

Information on RAI administration is provided in Table 3. Of the total cohort, 79% received RAI ablation/therapy, with a median dose of 3.7 GBq. The RAI ablation rate was significantly higher for T2 (94%) than for T1 patients (70%, *P* < 0.05). Overall, more patients with positive margins received RAI (92%) than patients with clear margins (77%, *P* < 0.001). A significant difference was also found for T1 patients (86% versus 68%, *P*=0.004), but not for T2 patients (98% versus 93%, *P*=0.180). No significant dosage difference was found between any of the subgroups with a median dose of 3.7 GBq.

### 3.2. Persistent/Recurrent Disease

There was a wide range of follow-up periods in the whole cohort, from 3 to 20 years, but there was no statistically significant difference in the follow-up periods between the positive margin group and the clear margin group. As outlined in the Methods section, we used strict definitions for disease-free status, persistent disease, and recurrent disease.

Outcome data are shown in Table 4. The response to initial management was assessed at 12 months (plus or minus 3 months) after completion of surgery with or without RAI therapy. For the entire cohort, a disease-free status was found in 97%, while 3% of patients had persistent disease. Persistent disease was significantly higher in the positive margin group (8%) than in the clear margin group (2%, *P*=0.001). However, in patients with positive margins who were disease-free after the initial management, the recurrence rate was 1%, which is similar to the recurrence rate in patients with clear margins.

This was confirmed when we performed a multivariate regression analysis for all patients (combined with and without RAI) when comparing disease-free versus persistent disease status (Table 5) and disease-free versus recurrent disease status (Table 6). We found that positive microscopic surgical margins (hazard ratio (HR) = 2.952) and nodal metastases (N1a and N1b; HR 5.728 and 25.977, respectively) were independent risk factors for persistent disease, while sex, age, T staging, and histology were not risk factors for persistent disease (*P*=0.113, 0.306, 0.969, and 0.998, respectively) (Table 5). We also found that only nodal involvement (N1a and N1b; HR 7.815 and 17.108, respectively) was an independent risk factor for recurrent disease, while age, sex, T staging, histology, and positive margins were not risk factors for recurrence (Table 6).

Thus, as nodal involvement was a significant risk factor for both persistent disease and disease recurrence, it was shown that the degree of significance increased with nodal staging. For example, N1b stage was 26 times more likely to have persistent disease than being disease-free versus approximately 6 times for N1a, and this stage (N1b) was 17 times more likely to have disease recurrence versus approximately 8 times in N1a.

**Figure 1:**
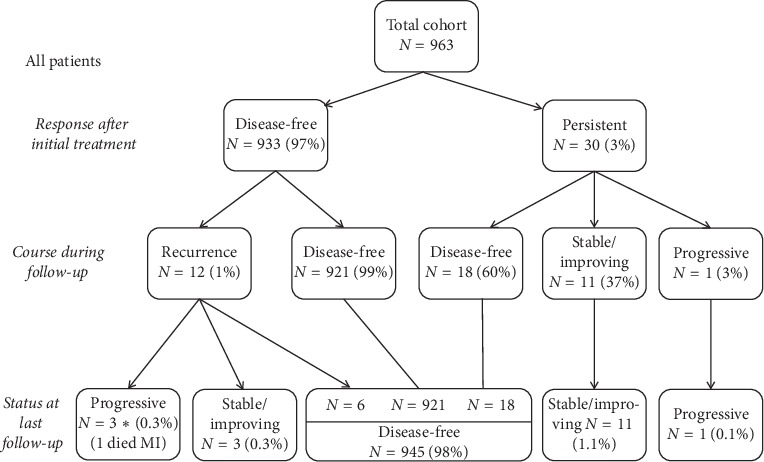
Flow chart demonstrating the stratification of all patients after initial therapy, during follow-up, and at last follow-up.

**Figure 2:**
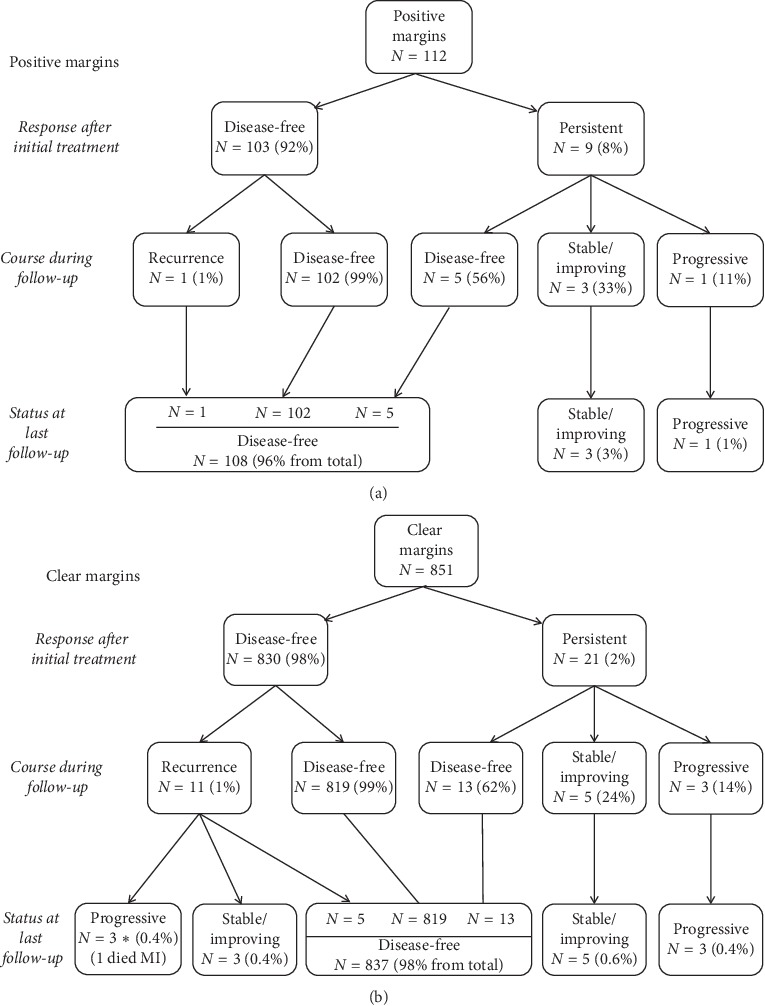
(a) Flow chart demonstrating the stratification of patients with positive margins after initial therapy, during follow-up, and at last follow-up. (b) Flow chart demonstrating the stratification of patients with clear margins after initial therapy, during follow-up, and at last follow-up.

At the time of the last available follow-up, 98% of the total cohort was disease-free, 1.4% had persistent stable/improving disease, and 0.4% had persistent progressive disease (one patient died because of myocardial infarction, which was likely secondary to his metastatic disease; there were 12 patients with disease recurrence: eight had both biochemical and structural recurrence, three had biochemical only, and one had structural only disease recurrence) (Figure 1). A more detailed analysis of patient trajectories is shown in Figure 2(a) (the disease course in the positive margin group) and Figure 2(b) (the disease course in the clear margin group). Regarding patients who had persistent disease after initial treatment, 56% became disease-free in the positive margin group and 62% in the clear margins group at the last follow-up or later in the course. The fraction of patients with stable and progressive disease was also very similar between the two groups; i.e., microscopic positive margin was not a risk factor for the last available follow-up disease status stratification whether progressing, stable, or disease-free state.

## 4. Discussion

In this study, we investigated whether microscopic positive margins have a significant impact on disease outcome in patients with well-differentiated early-stage thyroid cancer. In patients who had undergone total thyroidectomy for T1-T2 DTC, we found that microscopic positive margins were present in 12% of the specimens. This was associated with an increased rate in persistent disease but not with an increased risk of disease recurrence. While microscopic positive margins were an independent risk factor only for persistent disease, nodal involvement was an independent risk factor for both persistent and recurrent disease.

In our study, we applied strict definitions for disease-free, persistent, and recurrent disease. We highlight that persistent, recurrent, and progressive disease could be either structural, biochemical, or a combination of both.

Additionally, we required a disease-free period documented by thyroid cancer markers normalization before diagnosing disease recurrence. In most studies, the persistence and recurrence of DTC after initial treatment have been indistinctly defined with no clear separation. Few studies have investigated the difference between persistent and recurrent disease in terms of disease outcomes and survival rates [13–15]. In a recent study, Sapuppo et al. [13] emphasized the importance of this distinction and defined each disease status separately. They concluded that patients with DTC who are not cured after initial treatment are more prone to have persistent disease and have worse outcomes than recurrent disease.

The surgical margin is defined as the surface of the surgical specimen, whether it is the outer aspect of the thyroid gland and/or the inked edge of the specimen. It is well known that the thyroid “capsule” is not an anatomically defined structure, and it can be microscopically incomplete or absent [16]. To determine the microscopic marginal status, the pathologist needs to determine the relationship of the tumor with the inked edge. If the tumor extends to the inked surgical margin, it has a positive surgical margin which does not correlate with incomplete excision. In other words, a tumor can extend to the inked margin and not be extrathyroidal. Similarly, a tumor can extend beyond the thyroid tissue (extrathyroidal extension) but not extend to the inked specimen margin. This will depend on how much tissue is removed surgically beyond the thyroid gland. As stated in the 2016 ATA guidelines, the status of the resection margins should be reported as “involved” or “uninvolved” with the tumor in the pathology report as positive margins are generally associated with an intermediate or high risk for recurrence [9]. The presence of a macroscopic (gross) extrathyroidal extension and incomplete tumor resection are important poor prognostic factors significantly affecting recurrence rates [9]. Previous studies investigating the microscopic positive margin status on patient outcomes and providing treatment recommendations have been scarce, especially in the early stages of the disease. Some studies have used variable criteria for patient selection, while others did not separate microscopic resection margin involvement from the gross residual tumor in addition to variable definitions and sometimes the lack of separation between persistent disease status versus disease recurrence among different studies. These nuanced differences between studies may result in different conclusions and subsequently variable management recommendations. Below we discuss some of these studies in more detail.

Kluijfhout et al. [7] recently reported a total of 684 patients with well-differentiated T1 and T2 thyroid cancers smaller than 4 cm. They found that 11% of these patients had microscopic positive margins on pathology examination and that this finding alone did not increase the chance of recurrence. In their study, recurrent disease was categorized as local thyroid bed recurrence, lymph node recurrence, or distant metastases confirmed by imaging or biopsy. Unlike our study, there was no clear separation between persistent and recurrent disease. Wang et al. [11], in a retrospective study reviewing 2,616 patients, found that 10% of DTC patients had positive microscopic surgical margins after excluding patients with gross residual or metastatic disease and patients with less than a total thyroidectomy. In this study, microscopic positive margin was not a significant predictor of local failure (recurrence). Local recurrence was mainly structural and confirmed by imaging and the biopsy of lesions in the thyroid bed only, with no specific time frame from initial surgery. The authors recommended that highly selected patients with microscopic positive margins may be observed without RAI. Tsang et al. [12] studied the impact of the type of surgery, RAI, and external beam radiation therapy on different stages of DTC with higher rates of the poorly differentiated type. Patients with a high risk of disease at that time, including microscopic and macroscopic residual disease, were treated with adjuvant radiation therapy to the neck. They found that microscopic residual disease was present in 54% of patients and that it was not associated with local recurrence. Microscopic disease in this study included pathologic positive margins and when cancer was shaved off adjacent structures. The caveat to this study was the broader definition of microscopic disease and more aggressive therapeutic measures at that time, which would have potentially affected the outcomes.

In another study, Hong et al. [10], investigated the relevance of positive margins after high-dose adjuvant RAI ablation in patients with DTC. They found that 6.1% of patients had positive microscopic surgical margins and that those patients had a significantly higher incidence of early recurrence (within 1 year) and lower disease-free survival and no difference in late recurrence rate (after one year). These findings are in line with the present study. However, it should be noted that early recurrence in the study by Hong et al. could actually have been persistent disease. All in all, these studies illustrate the critical importance of clear definitions when comparing studies on persistent versus recurrent disease in patients with DTC.

An additional consideration is that the above studies did not include biochemical parameters to examine persistent versus recurrent disease. The structural evidence of persistent/recurrent disease is usually limited by the spatial resolution of the imaging modality used or by the minimum size that can be detected by clinical examination. Having a very sensitive biochemical thyroid cancer marker can significantly enhance the detectability of persistent/recurring disease that is microscopical or too small to be detected clinically or by imaging. Multiple studies [17–20] found that approximately 20% of patients who have no clinical evidence of disease and undetected TG, regardless of the risk of recurrence, will present with stimulated TG levels above 2 ng/ml whether after recombinant human thyrotropin (rhTSH) or thyroid hormones withdrawal. In those patients with positive biochemical markers, structural persistent/recurrent disease can be identified on imaging in only one-third of the patients. Thus, accounting for both biochemical and structural parameters allows for a more sensitive screening.

In our cohort of relatively low-risk patients with only T1 and T2 tumor staging and no metastatic disease, 79% received RAI ablation/therapy, with a median dose of 3.7 GBq. Significantly more patients with positive margins received RAI (92%) than patients with clear margins (77%), with no significant difference in the median dose. This difference was also found for T1 patients (86% versus 68%) but not for T2 patients (98% versus 93%) as T2 patients already had a higher RAI ablation rate (94%) than T1 patients (70%) whether they had positive or clear margins. This higher RAI ablation rate in the positive margin group was directly related to the contemporaneous guidelines and local practices during the long period of data collection covered in our study (1995–2013) and could by itself have affected the disease outcome. Thus, it is possible that having this higher RAI ablation rate might explain the lack of difference in disease recurrence between microscopic positive and negative margins and also the lack of effect on disease progression, persistence, or disease-free status in the last available follow-up visit. The small number of patients with persistent/recurrent disease who had positive margins precludes further analysis of the RAI effect in each subgroup.

Today, the most recent ATA guidelines in 2015 [9] do not routinely recommend RAI ablation for low-risk patients, but only for selected intermediate and high-risk patients, which in turn could have an impact of future disease outcomes. Thus, at present, it is not clear if the omission of RAI in patients with positive microscopic margins can affect the recurrence or persistence risk. Our study showed a higher rate of persistent disease for positive margins, but this did not affect the recurrence rate or the final outcome. We suggest that future studies need to assess if there should be a more selective approach to offering RAI ablation for patients with positive margin specimens who have early-stage disease with no distant metastases or gross extrathyroidal extension. In the meantime, due to the significantly higher rates of persistent disease within the first year in our study, we suggest close monitoring by regular TG measurements and close follow-up by ultrasound may be an appropriate recommendation for early-stage DTC patients with positive microscopic surgical margins. This needs to be further studied preferably with prospective studies that assess the role of RAI in patients with positive surgical margins and its effect on disease recurrence.

Due to the retrospective nature of this study with up to a 20-year maximum follow-up period, there are several inherent limitations. First, this was a single-center study extended over a long period of time and thus was subjected to the local referral pattern and patient population. Second, there was a lack of histopathology report consistency regarding the margin status and staging in general. Some of the older reports did not mention the margin status and were considered as negative in the database. They also did not provide information on angioinvasion or multifocality, which could potentially be interesting characteristics in relation to positive versus negative margin status and may also affect recurrence risk. Third, our results were influenced by inherent selection bias by the treating physicians and surgeons according to the concurrent guidelines, thereby leading to higher rates of RAI ablation in patients with positive microscopic surgical margins, which in turn might have affected the disease outcomes.

## 5. Conclusions

In our cohort of 963 adult patients with T1 and T2 DTC, 12% had positive microscopic surgical margins on histopathology reports. Presence of positive microscopic surgical margins was independently associated with a significantly higher rate of persistent disease, but not recurrent disease. Given these findings, the utility of radioactive iodine in reducing recurrence risk for early-stage differentiated thyroid carcinoma with microscopic surgical margins should be examined in future studies. While awaiting these studies, we suggest that a close follow-up of biomarkers and occult residual cancerous lesions would be appropriate, especially in the first year.

## Figures and Tables

**Table 1:** Clinical characteristics.

Clinical characteristics	All patients *n* = 963	Positive margins *n* = 112 (12%)	Clear margins *n* = 851 (88%)	*P* value^*∗*^
Male	152 (16%)	16 (14%)	136 (16%)	NS
Female	811 (84%)	96 (86%)	715 (84%)	
Age at diagnosis (years, mean ± SD)	46 ± 13	47 ± 14	46 ± 13	NS
Initial thyroid surgical management				
Total thyroidectomy	627 (65%)	67 (60%)	560 (66%)	NS
Two-part total thyroidectomy	336 (35%)	45 (40%)	291 (34%)	
Initial neck dissection surgical management				
Central neck dissection lateral	86 (9%)	7 (6%)	79 (9%)	NS
Neck dissection mediastinal	33 (4%)	3 (3%)	30 (4%)	
Dissection	1 (0.1%)	0 (0%)	1 (0.1%)	
Unspecified neck dissection^*∗∗*^	2 (0.2%)	0 (0%)	2 (0.2%)	
Histology				
Papillary	915 (96%)	108 (96%)	807 (95%)	NS
Follicular	48 (4%)	4 (4%)	44 (5%)	
Staging				
T1	603 (63%)	58 (52%)	545 (64%)	0.012
T2	360 (37%)	54 (48%)	306 (36%)	
Nx/N0	821 (85%)	86 (77%)	735 (86%)	0.024
N1a/N1b [N1a, N1b]	142 (15%) [116, 23	26 (23%)	116 (14%)	

Data are presented as number (percentages), unless otherwise specified. ^*∗*^Positive versus clear margins. ^*∗∗*^Unspecified neck dissection: neck dissection was done but extent was not specified in the operative report.

**Table 2:** Comparing T1 versus T2 patients with positive margins.

	Positive margins *n* = 112	*P* value
T1	58 (52)	0.026
T2	54 (48%)	

**Table 3:** RAI treatment: positive versus clear margins.

	All patients *n* = 963	Positive margins *n* = 112	Clear margins *n* = 851	*P* value^*∗*^
Initial RAI ablation				
Received RAI	761 (79%)	103 (92%)	658 (77%)	<0.001
RAI dose (GBq)	3.7 (1.1–7.4)	3.7 (1.8–5.5)	3.7 (1.1–7.4)	

T1 patients (n = 603)				
Received RAI	421 (70%)	50 (86%)	371 (68%)	0.004
RAI dose (GBq)	3.7 (1.1–5.7)	3.7 (1.8–5.5)	3.7 (1.1–5.7)	

T2 patients (n = 360)				
Received RAI	340 (94%)^#^	53 (98%)^#^	287 (93%)^#^	0.180
RAI dose (GBq)	3.7 (1.1–7.4)	3.7 (3.7–5.5)	3.7 (1.1–7.4^*∗∗*^)	

Data are presented as number (percentage) or median (range). ^*∗*^Positive versus clear margins. ^*∗∗*^Patient was treated with RAI in 1996, unexplained high dose. ^#^*P* < 0.05 for comparison with fraction of patients receiving RAI in T1 patients.

**Table 4:** Outcomes, positive versus clear margins, T1 and T2 stage.

	Total *n* = 963	Positive margins *n* = 112	Clear margins *n* = 851	*P* value^*∗*^
Follow-up				
Years of follow-up median (range)	7 (3–20)	6 (3–19)	7 (3–20)	0.570

After initial management				
Disease free	933 (97%)	103 (92%)	830 (98%)	0.001
Persistent disease	30 (3%)	9 (8%)	21 (2%)	

During follow-up				
Recurrence	12 (1%)	1 (1%)	11 (1%)	0.760

At last visit				
Persistent disease	18 (2%)	4 (4%)	14 (2%)	0.240
Disease-free	945 (98%)	108 (96%)	837 (98%)	

Data are presented as number (percentages), unless otherwise specified. Recurrence percentage based on the number of disease free excluding the persistent disease. ^*∗*^Positive versus clear margins.

**Table 5:** Multivariate analysis of factors affecting disease persistence.

Risk factor	Hazard ratio (95% CI)	*P* value
Sex (men)	0.344 (0.092–1.288)	0.113
Age at diagnosis	0.984 (0.953–1.015)	0.306
T staging	0.984 (0.432–2.242)	0.969
Nx/0	Referent	
N1a	5.728 (2.274–14.424)	0.001
N1b	25.977 (9.412–71.696)	0.001
Follicular histology	0.000	0.998
Positive margins	2.952 (1.225–7.111)	0.016

*Note*. T1 is the referent for T2 and Nx/0 is the referent for N1a and N1b, papillary histology is the referent for follicular thyroid cancer, women was the referent for men, and clear margins were the referent for positive margins.

**Table 6:** Multivariate analysis of factors affecting disease recurrence.

Risk factor	Hazard ratio (95% CI)	*P* value
Sex (men)	1.426 (0.372–5.464)	0.605
Age at diagnosis	1.027 (0.984–1.071)	0.231
T staging	1.073 (0.307–3.745)	0.912
Nx/0	Referent	
N1a	7.815 (1.977–30.898)	0.003
N1b	17.108 (3.500–83.628)	0.001
Histology	0.000	0.998
Positive margins	0.526 (0.062–4.467)	0.556

*Note*. T1 is the referent for T2 and Nx/0 is the referent for N1a and N1b, papillary histology is the referent for follicular thyroid cancer, women was the referent for men, and clear margins were the referent for positive margins.

## Data Availability

The clinical data used to support the findings of this study have not been made available because of patients' confidentiality and privacy rules.
